# A systematic review and meta-analysis of the association between arterial carbon dioxide tension and patient outcomes after cardiac arrest

**DOI:** 10.3389/fmed.2025.1687522

**Published:** 2025-11-06

**Authors:** Dandi Wang, Zhimao Li, Gerui Qin, Liping Cai, Ting Zhang, Hui Zhang, Yecheng Liu, Huadong Zhu

**Affiliations:** 1 Emergency Department, State Key Laboratory of Complex Severe and Rare Diseases, Peking Union Medical College Hospital, Chinese Academy of Medical Science and Peking Union Medical College, Beijing, China; 2 Department of Family Medicine & Division of General Internal Medicine, State Key Laboratory of Complex Severe and Rare Diseases,Peking Union Medical College Hospital, Chinese Academy of Medical Sciences, Beijing, China; 3 Department of Medicine, State Key Laboratory of Complex Severe and Rare Diseases, Peking Union Medical College Hospital, Chinese Academy of Medical Science and Peking Union Medical College, Beijing, China; 4 Department of Health Care, State Key Laboratory of Complex Severe and Rare Diseases, Peking Union Medical College Hospital, Chinese Academy of Medical Science and Peking Union Medical College, Beijing, China

**Keywords:** cardiac arrest, neurological outcomes, carbon dioxide tension, mortality, meta-analysis

## Abstract

**Background and aims:**

Current international guidelines suggest that normocapnia should be targeted during the resuscitation phase following cardiac arrest. However, some studies propose that therapeutic hypercapnia might be a potential strategy to enhance cerebral perfusion and improve patient outcomes after cardiac arrest. However, few studies have explored the association between arterial carbon dioxide tension and the prognosis of patients after cardiac arrest. This systematic review and meta-analysis aims to evaluate the influence of arterial carbon dioxide tension on the prognosis of patients after cardiac arrest.

**Data sources:**

We searched MEDLINE, Embase, and Cochrane CENTRAL to identify studies that evaluated the association between the partial pressure of arterial carbon dioxide and outcomes after cardiac arrest. The primary outcome was the neurological status at the end of the follow-up period. The secondary outcomes included short-, mid-, and long-term mortality. The meta-analysis was conducted if statistical heterogeneity was low.

**Results:**

Twelve studies were included. Compared with normocapnia, hypercapnia was associated with lower in-hospital mortality (pooled OR 0.74, 95% CI 0.59–0.92). For neurological outcomes, hypercapnia was associated with less short favorable outcome (pooled OR 0.42, 95% CI 0.22–0.8).

**Conclusion:**

Among cardiac arrest patients, hypercapnia was associated with a reduction in in-hospital mortality and favorable neurological outcome compared to normocapnia.

**Systematic review registration:**

This systematic review was registered with PROSPERO (ID: CRD42025644636).

## Introduction

Although cardiopulmonary resuscitation may initially be successful, a large number of patients who survive cardiac arrest do not make it to hospital discharge, and those who do often suffer from severe neurological damage. After cardiac arrest, the subsequent hours and days are characterized by post-cardiac arrest syndrome, which includes a pronounced inflammatory reaction, failure of organ bioenergetics, and an elevated risk of morbidity and mortality.

The partial pressure of arterial carbon dioxide (PaCO_2_) is crucial for the regulation of cerebral blood flow and has anti-convulsive, anti-inflammatory, and anti-oxidant effects, making therapeutic hypercapnia a potential strategy to improve cerebral perfusion following cardiac arrest. Global guidelines recommend aiming for normocapnia in unconscious adults who have been resuscitated from out-of-hospital cardiac arrest (1). However, many studies have indicated that compared with patients who maintain normocapnia, those exposed to hypercapnia or hypocapnia may have a better neurological prognosis at 12 months (2, 3). However, some studies have presented opposing views. For instance, Ebner et al. (4) indicated that while PaCO_2_ often fluctuates after cardiac arrest, it is not related to neurological outcomes. In summary, there is currently no consensus on the optimal target for PaCO_2_ in patients with cardiac arrest.

This meta-analysis aims to compare the effects of different levels of hypercapnia on the prognosis of patients after cardiac arrest, providing new insights into the diagnosis and treatment of this population.

## Methods

The systematic review and meta-analysis protocol was prospectively registered in the PROSPERO database [CRD42025644636]. The study was performed in accordance with the Preferred Reporting Items for Systematic reviews and Meta-Analyses (PRISMA) guidelines.

### Eligibility criteria

Ovid MEDLINE (R), Ovid MEDLINE (R) in process and other non-indexed citations, Ovid Embase, CINAHL Plus with Full Text (EBSCO), and the Cochrane Central Register of Controlled Trials (CENTRAL) were searched to identify eligible studies published between database inception and June 2024. The reference lists of review articles, editorials, and citation lists in Scopus were also hand-searched to identify possible additional studies. The end date for the literature search was 1 June 2024. For papers that met the inclusion criteria but that did not report patient outcomes for the PaCO_2_ group, the authors were contacted to request the original data.

### Data search

The key search terms were “Heart Arrest” AND “Carbon Dioxide” AND “Outcomes Assessment.” The detailed search strategy is described in the appendix (Supplementary Table S1). We used the Medical Subject Headings database to identify synonyms, and no language restrictions were applied. For trials that were registered and completed but not yet published, we contacted the authors to obtain the aggregate results.

### Study selection and data extraction

Two authors (DDW, ZML) independently screened the articles for inclusion on the basis of their titles and abstracts, and reviewed potentially eligible full-text papers according to predefined criteria. We collected data on trial characteristics, demographics, intervention and control procedures, and review outcomes from the eligible studies. In trials with multiple study arms, only data from the arms relevant to this review were extracted. When crucial information was missing, we contacted the corresponding authors. For transparency, we explicitly categorized the exposure definitions used in the included studies. The majority of studies reported true arterial PaCO_2_ values obtained from arterial blood gas analysis. However, Inoue et al. (5) used a surrogate measure, obtaining blood samples from a femoral artery or vein, which we have indicated in Table 1.

**Table 1:** Characteristics of studies included in the meta-analysis.

Author ID	Year	Study design	Regions	Sampling time	Groups	No. of participants	Definition (mmHg)
Eastwood et al.	2016	RCT	New Zealand	ABG with worst oxygenation during first 24 h in ICU	Hypercapnia	42	50–55
					Normocapnia	41	35–45
					Hypocapnia	18	<35
Jakkula et al.	2018	RCT	Multicenter	ABG in 36 h in the intensive care unit	Hypercapnia	59	43.5–45
					Normocapnia	NA	NA
					Hypocapnia	61	33.75–35.25
Eastwood et al.	2023	RCT	Multicenter	ABG in 24-h period beginning at randomization.	Hypercapnia	829	50–55
					Normocapnia	839	35–45
					Hypocapnia	NA	NA
Schneider et al.	2013	Observational	New Zealand	ABG with worst oxygenation during first 24 h in ICU	Hypercapnia	6,705	>45
					Normocapnia	6,827	35–45
					Hypocapnia	1,220	<35
Robert et al.	2013	Observational	USA	All ABG’s during first 24 h in ICU	Hypercapnia	60	>50
					Normocapnia	63	30–50
					Hypocapnia	52	<30
Lee et al.	2014	Observational	Korea	All ABG’s between ROSC and end of TH	Hypercapnia	152	>45
					Normocapnia	17	35–45
					Hypocapnia	44	<35
Vaahersalo et al.	2014	Observational	Multicenter	All ABG’s during first 24 h in ICU	Hypercapnia	208	>45
					Normocapnia	31	35–45
					Hypocapnia	170	<35
Helmerhost et al.	2015	Observational	Netherland	ABG with worst oxygenation during first 24 h in ICU	Hypercapnia	1834	>45
					Normocapnia	2,288	35–45
					Hypocapnia	1,136	<35
Wang et al.	2015	Observational	China	First ABG after sustained ROSC	Hypercapnia	143	>50
					Normocapnia	146	30–50
					Hypocapnia	261	<30
Zhou et al.	2020	Observational	China	ABG during the first 72 h in the ICU	Hypercapnia	762	>45
					Normocapnia	1,088	35–45
					Hypocapnia	933	<35
Okada et al.	2022	Observational	Multicenter	PaCO2 within 24 h after ROSC	Hypercapnia	332	>45
					Normocapnia	111	35–45
					Hypocapnia	94	<35
Inoue et al.	2023	Observational	Japan	PCO2 on arrival (a femoral artery or vein)	Hypercapnia	195	>100
					Normocapnia	396	60–100
					Hypocapnia	187	<60

### Risk of bias assessment of individual studies

The 21-item Newcastle–Ottawa scale was used to assess the methodological quality of the studies included in this systematic review (6). The results were collated, and the accuracy was independently checked by two authors. To evaluate the risk of bias in the individual randomized controlled trials (RCTs), we used the revised uniform criteria of the Cochrane risk-of-bias tool for randomized trials, version 2. We assessed the certainty of evidence for each outcome using the Grading of Recommendations, Assessment, Development, and Evaluations (GRADE) approach (7). Any disagreements were resolved by discussion and referral to a third author (YCL) if necessary.

### Outcomes

The primary outcome was short-term favorable neurological outcomes. The neurological prognosis was described according to the Cerebral Performance Category (CPC), Glasgow Outcome Scale-Extended score, or modified Rankin Scale score. We defined the short-term favorable neurological outcome was defined as CPC 1–2 < 1 month. The secondary outcomes were long-term neurological outcomes, and short-term, mid-term, and long-term mortality. We defined the long-term favorable neurological outcome was defined as CPC 1–2 at 6–12 months, short-, mid-, and long-term mortality were defined as hospital mortality, 1-month mortality, and 6-month mortality, respectively.

### Data analysis

We used DerSimonian and Laird random-effects models to conduct the meta-analysis. We generated study weights using the inverse variance weighting method. The results are presented as relative risks and risk differences for dichotomous outcomes and as standardized mean differences for continuous outcomes, all with 95% confidence intervals (CIs) and 95% prediction intervals (RevMan, version 5.3, Cochrane Collaboration). For outcomes with substantial heterogeneity (I^2^ > 50%), pooled estimates were calculated using a random-effects model and are reported as exploratory with cautious interpretation.

### Heterogeneity

Studies were tested for heterogeneity using the I^2^ statistic. I^2^ values of <25, 25–50%, and >50% were considered to have low, moderate, and high heterogeneity between studies, respectively. We initially proposed to report a pooled estimate only when the I^2^ statistic was <50%. However, given that substantial heterogeneity was anticipated across observational studies, we additionally performed random-effects meta-analyses (DerSimonian–Laird and Hartung–Knapp–Sidik–Jonkman [HKSJ] adjustments) even when I^2^ exceeded 50%, and reported 95% prediction intervals to reflect the uncertainty of effect size distribution. In these cases, the “overall” estimates are presented as exploratory summaries, while interpretation focuses on directionality and robustness rather than precise point estimates. Narrative synthesis was also provided to complement these pooled results. We also considered differences in the follow-up times and variations in the PaCO_2_ cutoffs used to define normocapnia as potential sources of clinical heterogeneity among the studies. We planned to conduct a sensitivity analysis to determine the effects of these variations on the overall conclusion of the meta-analysis.

## Results

**Figure 1:**
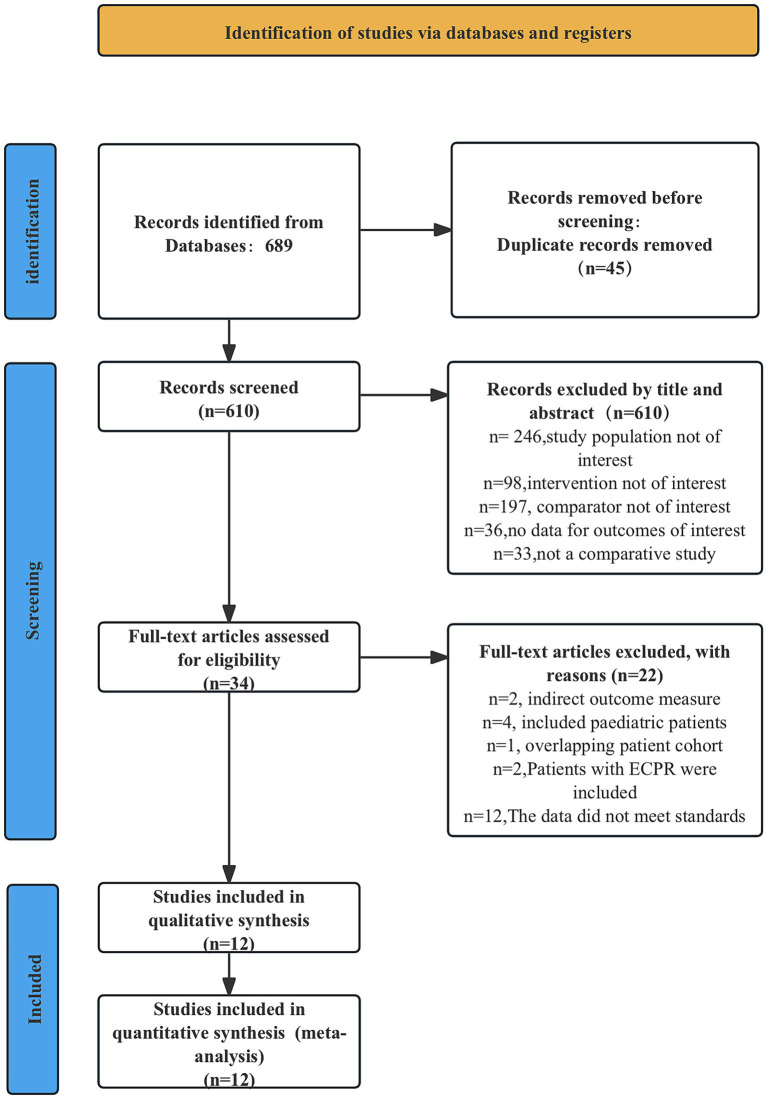
PRISMA flowchart illustrating the identification, screening, and inclusion of relevant literature. PRISMA, Preferred Reporting Items for Systematic reviews and Meta-Analyses.

Upon database searching, we identified a total of 689 records. Before reading, we removed 45 duplicate records, and based on the titles and abstracts of the articles, 610 were excluded. We carefully reviewed the remaining 34 articles and selected 12that met the inclusion criteria, including 3 RCTs (3, 8, 9) and 9 observational studies (3, 5, 10–16). Figure 1 shows the entire article screening process.

### Study characteristics

All 12 studies were published in the English language between 2013and 2024. The studies originated from Australia and New Zealand (combined), the United States, South Korea, China and the Netherlands, and collectively, they involved a total of 27,243 patients (ranging from 44 to 23,803 patients in each study). Table 1 presents the characteristics of each of the identified articles.

Among the 12 included studies, 11 reported arterial PaCO_2_ directly from arterial blood gas analysis, whereas 1 studies used surrogate measures. Specifically, 1 reported venous or unspecified PCO_2_ values on admission.

### Association between PaCO_2_ and neurological outcomes

A total of 8 studies reported neurological outcomes,4 of these studies compared the short neurological outcomes between patients with hypercapnia and those with normocapnia, as well as between those with normocapnia and those with hypocapnia.

**Figure 2:**
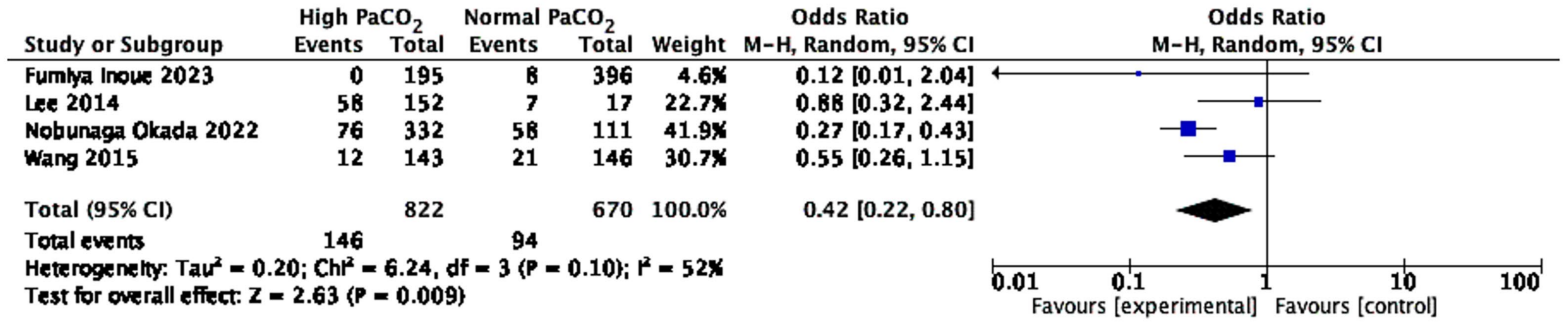
Forest plot of the comparison of short-term neurological outcomes between the hypercapnia group and the normocapnia group.

Compared with normocapnia, hypercapnia was associated with a significantly reduced odds of short favorable neurological outcome (OR 0.42, 95% CI 0.22–0.80), suggesting a better neurological prognosis (Figure 2). The study results exhibited moderate high heterogeneity (I^2^ = 52%). We conducted a sensitivity analysis on the aforementioned results (Supplementary Table S2) and found that after removing the study by Lee et al., which kept temperature of 33 °C ± 1 °C for 24 h. The results remained significant, with a substantial reduction in heterogeneity. However, there was no significant difference in the impact of PaCO_2_ level on the long-term central nervous system prognosis among the three groups of patients. Detailed results are presented in Supplementary Figures S3–S7.

### Association between PaCO_2_ and mortality

**Figure 3:**
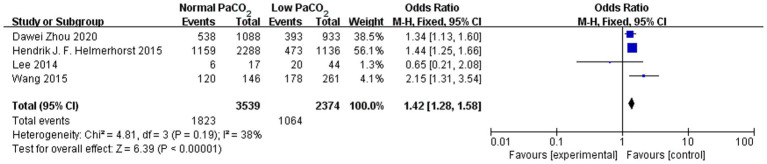
Forest plot of the comparison of mild-term mortality between the normocapnia group and the hypocapnia group.

Among the included studies, eight compared the mortality rates between different PaCO_2_ level. Hypercapnia and hypocapnia vs. normocapnia was associated with reduced in-hospital mortality (random-effects pooled OR 0.74, 95% CI 0.59–0.92; k = 6; I^2^ = 76%; OR 1.42, 95% CI 1.28–1.58 I^2^ = 38%; Figure 3). Detailed results are presented in Supplementary Figures S8–S12. Given the substantial heterogeneity in some comparisons (e.g., I^2^ = 76% for in-hospital mortality) these pooled estimates should be interpreted with caution.

## Discussion

In this meta-analysis, we showed that compared with patients with normocapnia, patients with hypercapnia were significantly associated with bad neurological status after cardiac arrest, especially in the short-term. Additionally, hypercapnia and hypocapnia may be associated with reduced short-term and mid-term mortality compared with normocapnia.

Among individuals who experience cardiac arrest, the main cause of death and long-term disability in survivors during the acute phase is brain injury, commonly referred to as post-cardiac arrest brain injury (or hypoxic–ischemic brain injury [HIBI]). Previous studies have proposed that PaCO_2_ is a major physiological regulator of cerebral vascular tone, with each 1 mmHg increase in PaCO_2_ leading to a 2 mL/100 g increase in cerebral blood flow. Furthermore, although it is not sufficient to assess patient outcomes, a multicenter phase 2 randomized trial showed that hypercapnia significantly reduced the release of neuron-specific enolase, a biomarker of brain injury (17–20). However, the aforementioned conclusions are not entirely consistent with the findings of the present study. A primary reason for this discrepancy may be that the included literature did not specify the severity of hypercapnia, and the possibility of extreme or uncontrolled hypercapnia cannot be excluded. Such pronounced hypercapnia could lead to severe respiratory acidosis, causing a significant decrease in intracellular pH, which may in turn inhibit enzymatic function, exacerbate cerebral edema, and potentially increase intracranial pressure, ultimately resulting in deleterious effects.

The present study has some limitations that should be considered. First, a limited number of RCTs were included in the analysis; the analysis primarily included observational studies, which may explain the heterogeneity in our results. Although we used HKSJ adjustments and prediction intervals to mitigate the impact of heterogeneity, these pooled results should be interpreted cautiously and considered exploratory rather than definitive.

Additionally, among the three included RCTs, different carbon dioxide cutoffs were used. Although the sensitivity analysis attempted to remove these articles without significantly affecting the results, we still consider this to be one of the reasons for the high heterogeneity. Second, in the included literature, the timing of ABG sampling varied. Moreover, hypothermia treatment may be used in patients with cardiac arrest, and different temperatures can affect the measured PaCO_2_. The consistency of results across adjusted and unadjusted estimates suggests that our conclusions are robust, although residual confounding remains possible.

Finally, only a small number of articles graded hypercapnia, so we did not obtain an exact PaCO_2_ range that was associated with reduced patient mortality and improved neurological outcomes. In light of these limitations, additional studies are needed in the future to validate the conclusions of this article.

## Conclusion

In patients with cardiac arrest, hypercapnia was associated with reduced in-hospital mortality, but showed no clear benefit in short-term neurological outcomes and no difference in long-term outcomes. One result suggested a possible increase in 1-month mortality. These findings highlight the complexity of CO_2_ management after resuscitation and should be validated in future large-scale RCTs.

## Data Availability

The original contributions presented in the study are included in the article/Supplementary material, further inquiries can be directed to the corresponding authors.
