# Supplementary material for: A systematic review and meta-analysis of the association between arterial carbon dioxide tension and patient outcomes after cardiac arrest
Source: Front Med (Lausanne). 2025 Nov 6;12:1687522. doi: 10.3389/fmed.2025.1687522 (PMC12630108; doi:10.3389/fmed.2025.1687522)
Supplement: Supplementary file 1 [file Data_Sheet_1.docx]

# Supplementary appendix

## Table S1. PRISMA Checklist

| **Section/topic** | **#** | **Checklist item** | **Reported on page #** |
| --- | --- | --- | --- |
| **TITLE** | | |  |
| Title | 1 | A systematic review and meta-analysis of the association between arterial carbon dioxide tension and patient outcomes after cardiac arrest | 1 |
| **ABSTRACT** | | |  |
| Structured summary | 2 | Provide a structured summary including, as applicable: background; objectives; data sources; study eligibility criteria, participants, and interventions; study appraisal and synthesis methods; results; limitations; conclusions and implications of key findings; systematic review registration number. | 3 |
| **INTRODUCTION** | | |  |
| Rationale | 3 | Describe the rationale for the review in the context of what is already known. | 5 |
| Objectives | 4 | Provide an explicit statement of questions being addressed with reference to participants, interventions, comparisons, outcomes, and study design (PICOS). | 5 |
| **METHODS** | | |  |
| Protocol and registration | 5 | Indicate if a review protocol exists, if and where it can be accessed (e.g., Web address), and, if available, provide registration information including registration number. | 6 |
| Eligibility criteria | 6 | Specify study characteristics (e.g., PICOS, length of follow-up) and report characteristics (e.g., years considered, language, publication status) used as criteria for eligibility, giving rationale. | 6 |
| Information sources | 7 | Describe all information sources (e.g., databases with dates of coverage, contact with study authors to identify additional studies) in the search and date last searched. | 6 |
| Search | 8 | Present full electronic search strategy for at least one database, including any limits used, such that it could be repeated. | 6 |
| Study selection | 9 | State the process for selecting studies (i.e., screening, eligibility, included in systematic review, and, if applicable, included in the meta-analysis). | 6-7 |
| Data collection process | 10 | Describe method of data extraction from reports (e.g., piloted forms, independently, in duplicate) and any processes for obtaining and confirming data from investigators. | 7 |
| Data items | 11 | List and define all variables for which data were sought (e.g., PICOS, funding sources) and any assumptions and simplifications made. | 7 |
| Risk of bias in individual studies | 12 | Describe methods used for assessing risk of bias of individual studies (including specification of whether this was done at the study or outcome level), and how this information is to be used in any data synthesis. | 7-8 |
| Summary measures | 13 | State the principal summary measures (e.g., risk ratio, difference in means). | 8 |
| Synthesis of results | 14 | Describe the methods of handling data and combining results of studies, if done, including measures of consistency (e.g., I^2^) for each meta-analysis. | 8-9 |

| **Section/topic** | **#** | **Checklist item** | **Reported on page #** |
| --- | --- | --- | --- |
| Risk of bias across studies | 15 | Specify any assessment of risk of bias that may affect the cumulative evidence (e.g., publication bias, selective reporting within studies). | 9 |
| Additional analyses | 16 | Describe methods of additional analyses (e.g., sensitivity or subgroup analyses, meta-regression), if done, indicating which were pre-specified. | 9 |
| **RESULTS** | | |  |
| Study selection | 17 | Give numbers of studies screened, assessed for eligibility, and included in the review, with reasons for exclusions at each stage, ideally with a flow diagram. | 9-10 |
| Study characteristics | 18 | For each study, present characteristics for which data were extracted (e.g., study size, PICOS, follow-up period) and provide the citations. | 10 |
| Risk of bias within studies | 19 | Present data on risk of bias of each study and, if available, any outcome level assessment (see item 12). | 10 |
| Results of individual studies | 20 | For all outcomes considered (benefits or harms), present, for each study: (a) simple summary data for each intervention group (b) effect estimates and confidence intervals, ideally with a forest plot. | 10, 19 |
| Synthesis of results | 21 | Present results of each meta-analysis done, including confidence intervals and measures of consistency. | 11-12 |
| Risk of bias across studies | 22 | Present results of any assessment of risk of bias across studies (see Item 15). | 10, 23-25 |
| Additional analysis | 23 | Give results of additional analyses, if done (e.g., sensitivity or subgroup analyses, meta-regression [see Item 16]). | 12 |
| **DISCUSSION** | | |  |
| Summary of evidence | 24 | Summarize the main findings including the strength of evidence for each main outcome; consider their relevance to key groups (e.g., healthcare providers, users, and policy makers). | 13-14 |
| Limitations | 25 | Discuss limitations at study and outcome level (e.g., risk of bias), and at review-level (e.g., incomplete retrieval of identified research, reporting bias). | 16 |
| Conclusions | 26 | Provide a general interpretation of the results in the context of other evidence, and implications for future research. | 14-15 |
| **FUNDING** | | |  |
| Funding | 27 | Describe sources of funding for the systematic review and other support (e.g., supply of data); role of funders for the systematic review. | 17 |

## *From:* Moher D, Liberati A, Tetzlaff J, Altman DG, The PRISMA Group (2009). Preferred Reporting Items for Systematic Reviews and Meta-Analyses: The PRISMA Statement. PLoS Med 6(6): e1000097. doi:10.1371/journal.pmed1000097

## Table S2. Systematic Literature Search

**Database(s): PubMed**

**Search Strategy:**

| **#** | **Searches** | **Results** |
| --- | --- | --- |
| 1 | (("heart arrest"[Title/Abstract] OR "cardiac arrest"[Title/Abstract] OR "in-hospital cardiac arrest"[Title/Abstract] OR "out-of-hospital cardiac arrest"[Title/Abstract] OR "cardiorespiratory arrest"[Title/Abstract] OR "cardiopulmonary resuscitation"[Title/Abstract] OR "Asystole"[Title/Abstract] OR "Asystoles"[Title/Abstract]) AND ("carbon dioxide"[Title/Abstract] OR "hypercarbia"[Title/Abstract] OR "hypocarbia"[Title/Abstract] OR "normocarbia"[Title/Abstract] OR "dyscarbia"[Title/Abstract] OR "hypercapnia"[Title/Abstract] OR "hypocapnia"[Title/Abstract] OR "normocapnia"[Title/Abstract] OR "PaCO2"[Title/Abstract] OR "partial pressure of carbon dioxide"[Title/Abstract]) | |
| 2 | ("outcomes assessment"[Title/Abstract] OR "survival"[Title/Abstract] OR "neurological outcome"[Title/Abstract] OR "prognosis"[Title/Abstract] OR "cerebral performance category"[Title/Abstract])) | |
| 3 | NOT ("case reports"[Publication Type] OR "Comment"[Publication Type] OR "Letter"[Publication Type]) | |
| 4 | 1 AND 2 AND 3 | 251 |

**Database(s): Embase**

**Search Strategy:**

| **#** | **Searches** | **Results** |
| --- | --- | --- |
| 1 | ('heart arrest':ab,ti OR 'cardiac arrest':ab,ti OR 'in-hospital cardiac arrest':ab,ti OR 'out-of-hospital cardiac arrest':ab,ti OR 'cardiorespiratory arrest':ab,ti OR 'cardiopulmonary resuscitation':ab,ti OR 'asystole':ab,ti OR 'asystoles':ab,ti) | |
| 2 | ('carbon dioxide':ab,ti OR 'hypercarbia':ab,ti OR 'hypocarbia':ab,ti OR 'normocarbia':ab,ti OR 'dyscarbia':ab,ti OR 'hypercapnia':ab,ti OR 'hypocapnia':ab,ti OR 'normocapnia':ab,ti OR 'paco2':ab,ti OR 'partial pressure of carbon dioxide':ab,ti) | |
| 3 | ('outcomes assessment':ab,ti OR 'survival':ab,ti OR 'neurological outcome':ab,ti OR 'prognosis':ab,ti OR 'cerebral performance category':ab,ti) | |
| 4 | NOT ('comment':it OR 'letter':it OR 'case reports':it) | |
| 5 | 1 AND 2 AND 3AND4 | 373 |

**Database(s): Cochrane Library**

**Search date:**

| **#** | **Searches** | **Results** |
| --- | --- | --- |
| 1 | "heart arrest" OR "cardiac-arrest" OR "in-hospital cardiac arrest" OR "out-of-hospital cardiac arrest" OR "cardiorespiratory arrest" OR "cardiopulmonary resuscitation " OR "Asystole" OR "Asystoles" | |
| 2 | ("heart arrest"):ti,ab,kw OR ("cardiac-arrest"):ti,ab,kw OR ("in-hospital cardiac arrest"):ti,ab,kw OR ("out-of-hospital cardiac arrest"):ti,ab,kw OR ("cardiorespiratory arrest"):ti,ab,kw | |
| 3 | ("cardiopulmonary resuscitation "):ti,ab,kw OR ("Asystole"):ti,ab,kw OR ("Asystoles"):ti,ab,kw | |
| 4 | ("carbon-dioxide"):ti,ab,kw OR ("hypercarbia"):ti,ab,kw OR ("hypocarbia"):ti,ab,kw OR ("normocarbia"):ti,ab,kw OR ("dyscarbia"):ti,ab,kw | |
| 5 | ("hypercapnia"):ti,ab,kw OR ("hypocapnia"):ti,ab,kw OR ("normocapnia"):ti,ab,kw OR ("PaCO2"):ti,ab,kw OR ("partial pressure of carbon dioxide"):ti,ab,kw | |
| 6 | ("outcomes assessment"):ti,ab,kw OR ("survival"):ti,ab,kw OR ("neurological outcome"):ti,ab,kw OR ("prognosis"):ti,ab,kw OR ("cerebral performance category"):ti,ab,kw | |
| 4 | (1 OR 2 OR 3) AND (4 OR 5) AND (6) | 65 |

**Table S3. Summary of Results**

| **Outcome** | **No. Studies** | **Sample Size** | **OR (95% CI) with P value** | **I^2^, % (P value)** |
| --- | --- | --- | --- | --- |
| **Morality** |  |  |  |  |
| High PaCO_2_ vs. Normal PaCO_2_ (short term) | 6 | 9920 | 0.74 (0.59, 0.92) with 0.006 | 76 (0.0009) |
| High PaCO_2_ vs. Normal PaCO_2_ (long term) | 2 | 7771 | 1.03 (0.94, 1.13) with 0.52 | 48 (0.16) |
| Normal PaCO_2_ vs. low PaCO_2_ (short term) | 4 | 5913 | 1.42 (1.28, 1.58) with <0.00001 | 38 (0.19) |
| Normal PaCO_2_ vs. low PaCO_2_ (medium term) | 2 | 788 | 2.92 (1.08, 7.90) with 0.03 | 82 (0.02) |
| High PaCO_2_ vs. low PaCO_2_ (short term) | 4 | 5265 | 1.02 (0.81, 1.30) with 0.85 | 65 (0.04) |
| High PaCO_2_ vs. low PaCO_2_ (medium term) | 3 | 928 | 2.30 (0.35, 15.17) with 0.39 | 95 (<0.00001) |
| **Favorable neurological outcome** |  |  |  |  |
| High PaCO_2_ vs. Normal PaCO_2_ (short term) | 4 | 1492 | 0.42 (0.22, 0.80) with 0.009 | 52 (0.10) |
| High PaCO_2_ vs. Normal PaCO_2_ (long term) | 2 | 1786 | 0.98 (0.80, 1.19) with 0.81 | 0 (0.40) |
| Normal PaCO_2_ vs. low PaCO_2_ (short term) | 4 | 1256 | 0.68 (0.24, 1.89) with 0.56 | 87 (<0.0001) |
| High PaCO_2_ vs. low PaCO_2_ (short term) | 4 | 1408 | 0.39 (0.11, 1.44) with 0.16 | 90 (<0.00001) |
| High PaCO_2_ vs. low PaCO_2_ (long term) | 2 | 498 | 0.76 (0.53, 1.08) with 0.13 | 0 (0.53) |

Abbreviations: CI=Confidence Interval; OR=Odds Ratio.

**Note.** low PaCO2: <30/35 mmHg; Normal CO2: 0/35-45/50 mmHg; high CO2: >45/50 mmHg. Morality: Short term: Mortality at discharge; Medium term: Mortality at 1 month; Long term: Mortality at within 6 months. Favorable neurological outcome: Short term: Mortality at <1 month; Long term: Mortality at 6-12 months.

**Table S3.Sensitivity analysis by removing one study at a time.**

| **Study omitted** | **Pooled odds risk (95% CI)** | **Weight** | **Heterogeneity estimate (I^2^, %)** |
| --- | --- | --- | --- |
| **Favorable neurological outcome** |  |  |  |
| **High vs Normal (short term)** |  |  |  |
| Fumiya Inoue 2023 | 0.45 [0.23, 0.91] | 4.6% | 64% |
| Lee 2014 | 0.32 [0.22, 0.47] | 22.7% | 32% |
| Nobunaga Okada 2022 | 0.55 [0.31, 0.98] | 41.9% | 0% |
| Wang 2015 | 0.38 [0.15, 1.01] | 30.7% | 58% |
| **overall** | 0.42 [0.22, 0.80] |  | 46% |
|  |  |  |  |
| **High vs Normal (long term)** |  |  |  |
| G. Eastwood 2023 | 1.37 [0.61, 3.05] | 94.9% | - |
| Vaahersalo 2014 | 0.96 [0.78, 1.17] | 5.1% | - |
| **overall** | 0.98 [0.80, 1.19] |  | 0% |
|  |  |  |  |
| **High vs Low (short term)** |  |  |  |
| Fumiya Inoue 2023 | 0.63 [0.17, 2.31] | 12.8% | 92% |
| Lee 2014 | 0.24 [0.09, 0.66] | 28.1% | 76% |
| Nobunaga Okada 2022 | 0.42 [0.06, 2.86] | 30.1% | 90% |
| Wang 2015 | 0.29 [0.04, 2.42] | 29.0% | 93% |
| **overall** | 0.39 [0.11, 1.44] |  | 90% |
|  |  |  |  |
| **High vs Low (long term)** |  |  |  |
| Pekka Jakkula 2018 | 0.80 [0.53, 1.21] | 51.2% | - |
| Vaahersalo 2014 | 0.61 [0.29, 1.30] | 24.9% | - |
| **overall** | 0.76 [0.53, 1.08] |  | 0% |
|  |  |  |  |
| **Normal vs Low (short term)** |  |  |  |
| Fumiya Inoue 2023 | 0.97 [0.67, 1.41] | 24.9% | 35% |
| Lee 2014 | 0.47 [0.16, 1.43] | 20.9% | 89% |
| Nobunaga Okada 2022 | 0.64 [0.13, 3.23] | 27.2% | 91% |
| Wang 2015 | 0.63 [0.13, 3.06] | 27.0% | 91% |
| **overall** | 0.68 [0.24, 1.89] |  | 87% |
|  |  |  |  |
| **Mortality** |  |  |  |
| **High vs Normal (short term)** |  |  |  |
| Dawei Zhou 2020 | 0.79 [0.61, 1.01] | 24.5% | 72% |
| G. Eastwood 2023 | 0.69 [0.53, 0.89] | 26.6% | 68% |
| Glenn M. Eastwood 2016 | 0.75 [0.59, 0.95] | 7.5% | 80% |
| Hendrik J. F. Helmerhorst 2015 | 0.75 [0.53, 1.05] | 27.3% | 81% |
| Lee 2014 | 0.71 [0.59, 0.86] | 3.8% | 72% |
| Wang 2015 | 0.78 [0.62, 0.97] | 10.3% | 78% |
| **overall** | 0.74 [0.59, 0.92] |  | 76% |
|  |  |  |  |
| **High vs** **Normal (mid-term)** |  |  |  |
|  |  |  |  |
| **High vs Normal (long term)** | |  |  |
| G. Eastwood 2023 | 1.07 [0.96, 1.19] | 24.4% | - |
| Schneider 2013 | 0.91 [0.75, 1.11] | 75.6% | - |
| **overall** | 1.03 [0.94, 1.13] |  | 48% |
|  |  |  |  |
| **Normal vs low (short term)** |  |  |  |
| Dawei Zhou 2020 | 1.47 [1.28, 1.69] | 38.5% | 53% |
| Hendrik J. F. Helmerhorst 2015 | 1.40 [1.19, 1.65] | 56.1% | 58% |
| Lee 2014 | 1.43 [1.28, 1.60] | 1.3% | 35% |
| Wang 2015 | 1.39 [1.24, 1.55] | 4.1% | 0% |
| **overall** | 1.42 [1.28, 1.58] |  | 38% |
|  |  |  |  |
| **Normal vs low (mid-term)** |  |  |  |
| Fumiya Inoue 2023 | 1.73 [0.90, 3.32] | 51.8% | - |
| Nobunaga Okada 2022 | 4.77 [2.80, 8.13] | 48.2% | - |
| **overall** | 2.92 [1.08, 7.90] |  | 82% |
|  |  |  |  |
| **High vs low (short term)** |  |  |  |
| Dawei Zhou 2020 | 1.13 [0.88, 1.45] | 34.4% | 33% |
| Hendrik J. F. Helmerhorst 2015 | 1.06 [0.70, 1.61] | 38.1% | 68% |
| Lee 2014 | 0.95 [0.78, 1.17] | 9.7% | 56% |
| Wang 2015 | 1.04 [0.77, 1.40] | 17.8% | 76% |
| **overall** | 1.02 [0.81, 1.30] |  | 68% |
|  |  |  |  |
| **High vs low (mid-term)** |  |  |  |
| Fumiya Inoue 2023 | 0.94 [0.24, 3.70] | 32.0% | 89% |
| Nobunaga Okada 2022 | 5.21 [0.68, 39.83] | 34.6% | 90% |
| Pekka Jakkula 2018 | 2.59 [0.08, 84.21] | 33.4% | 97% |
| **overall** | 2.30 [0.35, 15.17] |  | 95% |

**Figure S1. Study risk of bias**


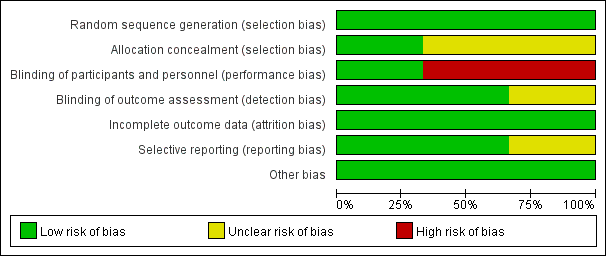


**Figure S2. Risk of bias summary**


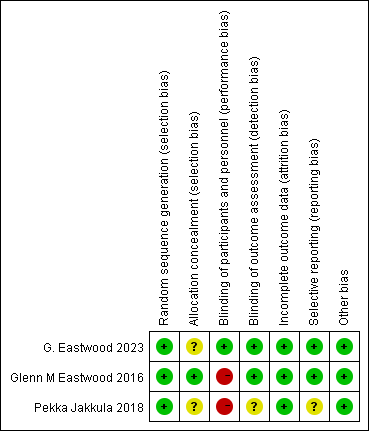


**Figure S3-7 Forest plot of the comparison of neurological outcomes between the different PaCO_2_ groups**


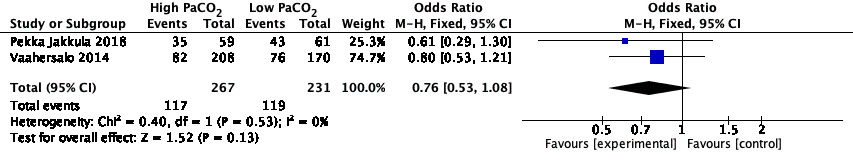


Figure S3 Forest plot of the comparison of long neurological outcomes between the hypercapnia group and the hypocapnia group.

**
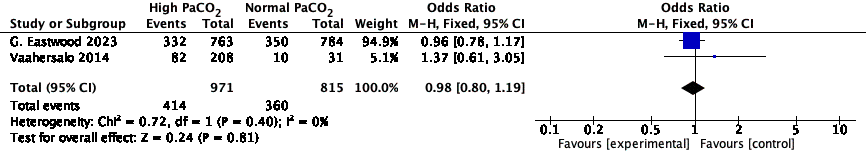
**

Figure S4 Forest plot of the comparison of long neurological outcomes between the hypercapnia group and the normocapnia group.


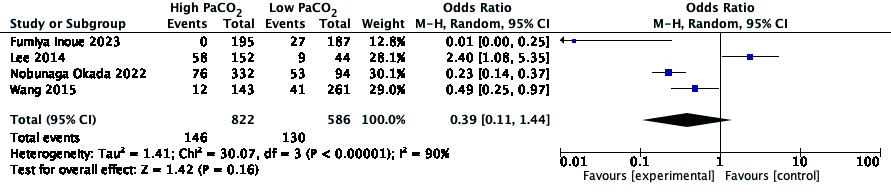


Figure S5 Forest plot of the comparison of short neurological outcomes between the hypercapnia group and the hypocapnia group.


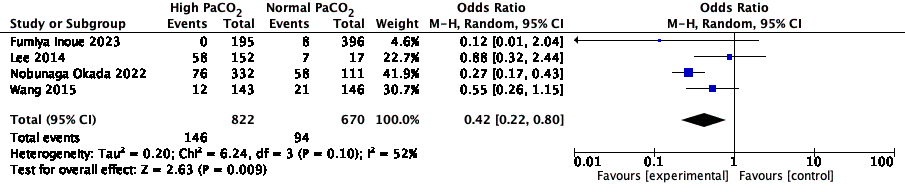


Figure S6 Forest plot of the comparison of short neurological outcomes between the hypercapnia group and the normocapnia group.


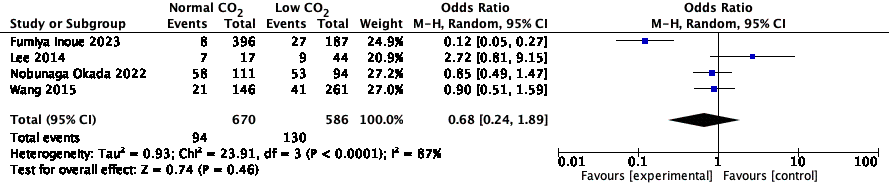


Figure S6 Forest plot of the comparison of short neurological outcomes between the hypocapnia group and the normocapnia group.

**Figure S8-12.Forest plot of the comparison of mortality between the different PaCO2 group.**


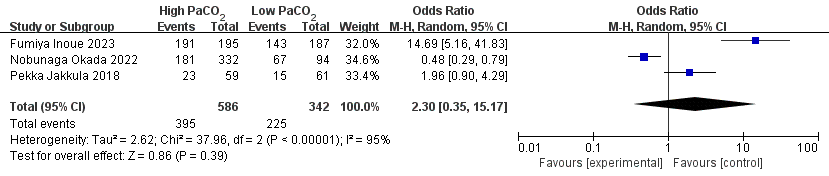


Figure S8 Forest plot of the comparison of medium motality between the hypercapnia group and the hypocapnia group.


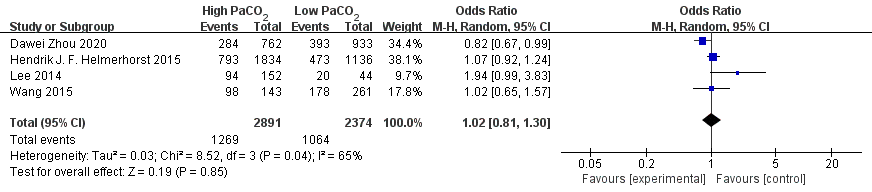


Figure S9 Forest plot of the comparison of short motality between the hypercapnia group and the hypocapnia group.


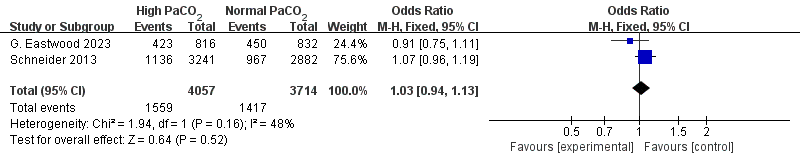


Figure S10 Forest plot of the comparison of long motality between the hypercapnia group and the normoapnia group.


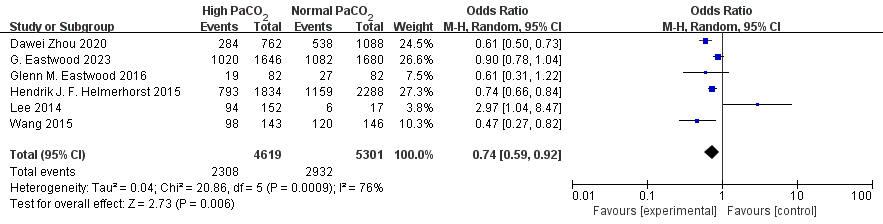


Figure S11 Forest plot of the comparison of short motality between the hypercapnia group and the normoapnia group.


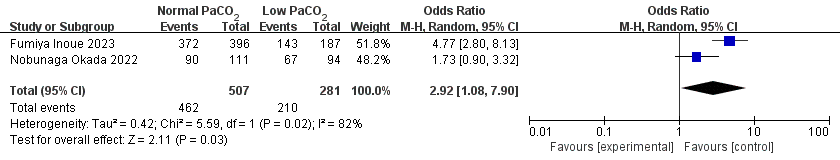


Figure S12Forest plot of the comparison of smedium motality between the hypocapnia group and the normoapnia group.
